# Dental Chemistry of the Mouth

**Published:** 1857-03

**Authors:** Geo. Watt


### DENTAL CHEMISTRY OF THE MOUTH.

BY GEO. WATT.

(Continued from page 186.)

MUCUS. The internal parts of the animal system which are directly connected with the external, are covered by a soft, velvety and very vascular tissue called the mucous membrane. This membrane is a continuation of the skin, and therefore, all parts of the system are enveloped in one and the same tegument, its internal and external portions differing very materially in regard to their exposed surfaces. The exposed surface of mucous membrane is covered by a delicate layer of epithelium. The membrane derives its name from the qualities of the fluid with which it is moistened, which fluid is a regular secretion, and serves to protect the delicate membrane from the action of irritants. This fluid is viscid, tenacious, sometimes colorless, but mostly turbid, of a faint yellow or grayish white color. It is secreted in but limited quantities when the membrane is in its normal condition; but in a state of irritation, the secretion is greatly increased.

It is difficult, if not impossible, to ascertain, precisely, either the physical or chemical properties of normal mucus, or even of abnormal. The normal can only be obtained in limited quantities; and the transition from normal to diseased mucus is so gradual and indefinite that it is impossible to define the precise point at which the change takes place. And besides, it is almost impossible to obtain mucus uncontaminated with other substances. It is little wonder then that Simon remarks, "Hence it is not very easy to form a distinct conception of what normal mucus really is."

We have said that the mucous membrane is a continuation of the skin; and, like the skin, it is constantly undergoing a desquamation, a separation of cuticular scales taking place in the one, a throwing off of epithelium cells in the other.

The specific gravity of healthy mucus, when fresh and recently secreted is greater than that of water, accordingly it sinks in that fluid, unless prevented by the presence of air bubbles or other causes.

It was once generally regarded as characteristic of mucus to float in water; and this test was regarded as sufficient to distinguish it from pus; but dried mucus, or fresh mucus from the intestines or urinary bladder, and even that from the bronchial and nasal cavities, when deprived of air bubbles, sinks rapidly in that fluid.

Fresh, liquid, transparent mucus, from the nasal or bronchial membrane, is found, by examination with the microscope, to consist of a fluid containing minute, rounded or elongated granular bodies, called mucus-corpuscles, and a more or less abundant supply of epithelium cells. According to Simon a finely granulated substance also pervades the fluid.

The mucus of the mouth is obtainable only in such limited quantities, besides being mingled with other substances, that great difficulty is experienced in obtaining a satisfactory knowledge of its chemical properties. Accordingly, nasal, bronchial, and intestinal mucus is generally selected in conducting experiments with this substance. Mucus being secreted by the same membrane, wherever found, is likely to have the same general properties.

Simon states that the epithelium cells "are not affected by the addition of water, or of dilute acids; they dissappear, however, under the influence of caustic alkalies or concentrated acids." These cells are also unaffected by the ordinary earthy and metallic salts. "The mucus-corpuscles," says Simon, "are very differently acted on. Dilute acetic, oxalic, and tartaric acid speedily deprive the capsules of the mucus-corpuscles of their granular appearance. The corpuscles themselves become round and transparent; the nuclei become apparent, the capsules at length dissappear, and the nuclei frequently divide into several granular bodies, so that in place of the mucus-corpuscles previously visible, there are at last only two, three or more rounded granules to be seen.

Simon states that the liquid portion of mucus always exhibits a decidedly alkaline reaction; but this is, at least, doubtful. Indeed there is scarce a doubt that morbid mucus often has a decided acid reaction. In analyses of mucus the acids are included in the extractive matters.

When distilled water is added to a clear fluid mucus a coagulation is perceived which soon is deposited as a fine granular precipitate. The same precipitate, more copious and tenacious, is thrown down by acetic, or almost any weak acid. This precipitate is not thrown down by the alkalies or their carbonates.

The precipitate thus obtained is supposed to be the characteristic chemical constituent of mucus, and is hence called *mucin*.

As mucin is soluble in solutions of the alkalies, and as it is precipitated by removing the alkali by an acid, or even by water, it seems rational to conclude that in normal mucus it is held in solution by an alkali.

The fluid portion of mucus also contains chloride of sodium, lactate of soda, and traces of a few other salts. We know but little, positively, of the contents of the mucus-corpuscles. Simon says, "In all probability they contain a fluid in addition to their nuclei. The fat that occurs in mucus is probably contained in the corpuscles; for no fat vesicles are generally observed in fresh mucus, but after the solution of the corpuscles by the addition of acetic acid, a few fat vesicles make their appearance.

It is probable that fat and albumen, though often found in normal mucus, are not essential constituents of it. By an analysis of nasal mucus Berzelius found in 1000 parts:—

| | |
|---|---|
| Water | 930. 7 |
| Mucin | 53. 3 |
| Alcohol extract and alkaline lactates | 3. 0 |
| Chlorides of Sodium and potassium | 5. 6 |
| Water extact with traces of albumen and phosphates | 3. 5 |
| Soda, combined with mucus | 3. 9 |
| | 1000. 0 |

We have no very thorough and reliable analysis of buccal mucus. It is almost impracticable to obtain mucus from this source in such condition and quantity as will be satisfactory. The annlysis of buccal mucus by Jacubowitsch, as quoted by Piggot, is as follows :—

| | | |
|---|---|---|
| Water | | 990. 02 |
| Solid matters :— | | |
| Organic matter soluble in alcohol | 1. 67 | |
| " " insoluble " | 2. 18 | |
| Fixed salts | 6. 13 | 9. 98 |
| | | 1000. 00 |

From the preceding observations we may conclude that mucus contains epithelium cells, mucus-corpuscles, mucin, small quantities of extractive matters, alkaline lactates, chlorides of sodium and potassium, a little phosphate of lime and carbonate of soda, usually a small quantity of fat, and sometimes a trace of albumen.

Simon adopts the following method of separating these substances in analysis:

"A known weight of mucus must be washed with distilled water and evaporated to dryness on the water bath. The residue must be finely triturated, and repeatedly extracted with boiling ether, in order to remove the fat; it must then be boiled in spirit of 0. 91 as long as any additional matter is dissolved. The spirituous solution must be evaporated to a small syrupy residue, and alcohol of 0. 85 added, in order to precipitate any dissolved mucin, caseous matter, water-extract, and pyin: the alcoholic solution, containing the alcohol-extract and lactates is also to be evaporated. The portion undissolved by boiling spirit of 0. 91, consists of mucin with cells, and traces of albumen, if the previous quantitative investigation has shown that this substance is present.

In order to determine the salts, a portion of the dried residue must be submitted to incineration. It is difficult to obtain a white ash in consequence of the fusion of the salts. The chlorides may be extracted with spirit; the residue must then be treated with acetic acid, in order to convert the carbonates, which have arisen from the incineration of the alkaline lactates, into acetates, which may be extracted with alcohol. Any thing that still remains is composed of phosphates and perhaps sulphates, in very minute quantity, together with traces of iron and silica."

Irritation of the membrane greatly increases the secretion of mucus. This is witnessed in the nasal and bronchial secretions during catarrhal affections, and is often as observable in inflammatory diseases of the mouth. In all these conditions, the mucus is considerably changed. At the onset of the attack, it is thinner than usual, but becomes thicker as the disease progresses; the mucus-corpuscles are increased in number and the epithelium cells diminished. Simon says, "the reaction continues alkaline; in fact, in most cases it is more strongly so than in the normal state." This is not according to my own observation; indeed, I have usually found an acid reaction in such cases; but my observations have not been sufficiently extensive to determine what is the rule. There is usually an excess of albumen and an increase of fat. According to our own observation, there is also a very perceptible increase of the soluble chlorides in the early stage of inflammation of the mucous membrane. Decomposition of these chlorides may take place from adventitious causes; and, if so, the nascent chlorine will form hydrochloric acid with the hydrogen of the water contained in the saliva. This may account, sometimes, for the acid reaction observed.

Much obscurity rests on this subject, and many difficulties lie in the way of its thorough investigation. We hope those who have the time and talent will undertake the task. In our next article, we will endeavor to describe the source and action of some of the principal acids which are likely to exert an injurious influence on the teeth.

